# Supplementary material for: Zileuton Attenuates Acute Kidney Injury in Glycerol-Induced Rhabdomyolysis by Regulating Myeloid-Derived Suppressor Cells in Mice
Source: Int J Mol Sci. 2025 Aug 28;26(17):8353. doi: 10.3390/ijms26178353 (PMC12428576; doi:10.3390/ijms26178353)
Supplement: Supplementary file 1 [file ijms-26-08353-s001.zip › ijms-3764071-supplementary.pdf]

Supplementary table S1. Primer Sequences Used for qPCR

Target	Forward (5’-3’)	Reverse (5’-3’)
Arginase -1	CATTGGCTTGCGAGACGTAGAC	GCTGAAGGTCTCTTCCATCACC
CX3CR 1	CACCATTAGTCTGGGCGTCT	GATGCGGAAGTAGCAAAAGC
CX3CL1	ATT GGA AGA CCT TGC TTT GG	GCC TCG GAA GTT GAG AGA GA
Mincle	ACCAAATCGCCTGCATCC	CACTTGGGAGTTTTTGAAGCAT C
TGF-β1	TGCGCTTGCAGAGATTAAAA	CGTCAAAAGACAGCCACTCA
iNOS	GAGACAGGGAAGTCTGAAGCA C	CCAGCAGTAGTTGCTCCTCTTC
PGC-1α	CTGACCACAAACGATGACCCTC	TGCGGTTGTGTATGGGACTTCT
TFAM	GCTGATGGGTATGGAGAAGGA G	TGAGCCGAATCATCCTTTGC
CPT1α	GGCATAAACGCAGAGCATTCCT G	CAGTGTCCATCCTCTGAGTAGC
ND1 (mtDNA)	ATCCTCCCAGGATTTGGAAT	ACCGGTAGGAATTGCGATAA
HMGB1	TGGCAAAGGCTGACAAGGCTC	GGATGCTCGCCTTTGATTTTGG
RAGE	GAA TAG TCG CTC CTG GTG GG	CAG CTA TAG GTG CCC TCA TCC
TLR4	ATCCCTGCATAGAGGTAGTTCC	GGTGGTGTAAGCCATGCCA
NLRP3	CATGTTGCCTGTTCTTCCAGAC	CGGTTGGTGCTTAGACTTGAGA
Caspase-1	TCAGCTCCATCAGCTGAAAC	AGTCCTGGAAATGTGCCATC
IL-1β	CTTCAGGCAGGCAGTATCACTC AT	TCTAATGGGAACGTCACACACC AG
IL-18	GCTGTGACCCTCTCTGAGAA	GGCAAGCAAGAAAGTGTCCT
GAPDH	GACATCAAGAAGGTGGTGAAG C	GAAGGTGGAAGAGTGGGAGTT
